# Rectal microbiota are coupled with altered cytokine production capacity following community-acquired pneumonia hospitalization

**DOI:** 10.1016/j.isci.2022.104740

**Published:** 2022-07-14

**Authors:** Robert F.J. Kullberg, Xanthe Brands, Augustijn M. Klarenbeek, Joe M. Butler, Natasja A. Otto, Daniël R. Faber, Brendon P. Scicluna, Tom van der Poll, W. Joost Wiersinga, Bastiaan W. Haak

**Affiliations:** 1 Center for Experimental and Molecular Medicine (CEMM), Amsterdam University Medical Centers - Location AMC, University of Amsterdam, Amsterdam, the Netherlands; 2 Department of Internal Medicine, BovenIJ Hospital, Amsterdam, the Netherlands; 3 Department of Applied Biomedical Science, Faculty of Health Sciences, Mater Dei Hospital, University of Malta, Malta; 4 Centre for Molecular Medicine and Biobanking, University of Malta, Malta; 5 Division of Infectious Diseases, Amsterdam University Medical Centers - Location AMC, University of Amsterdam, Amsterdam, the Netherlands

**Keywords:** Immunology, Microbiology, Microbiome

## Abstract

Human studies describing the immunomodulatory role of the intestinal microbiota in systemic infections are lacking. Here, we sought to relate microbiota profiles from 115 patients with community-acquired pneumonia (CAP), both on hospital admission and following discharge, to concurrent circulating monocyte and neutrophil function. Rectal microbiota composition did not explain variation in cytokine responses in acute CAP (median 0%, IQR 0.0%–1.9%), but did one month following hospitalization (median 4.1%, IQR 0.0%–6.6%, p = 0.0035). Gene expression analysis of monocytes showed that undisrupted microbiota profiles following hospitalization were associated with upregulated interferon, interleukin-10, and G-protein-coupled-receptor-ligand-binding pathways. While CAP is characterized by profoundly distorted gut microbiota, the effects of these disruptions on cytokine responses and transcriptional profiles during acute infection were absent or modest. However, rectal microbiota were related to altered cytokine responses one month following CAP hospitalization, which may provide insights into potential mechanisms contributing to the high risk of recurrent infections following hospitalization.

## Highlights

• Rectal microbiota are disrupted at hospitalization for CAP and one month thereafter
• No variation in cytokines is explained by gut microbiota in the acute phase of CAP
• Following recovery, gut microbiota are linked with variation in cytokine responses

## Graphical abstract

**Figure:**
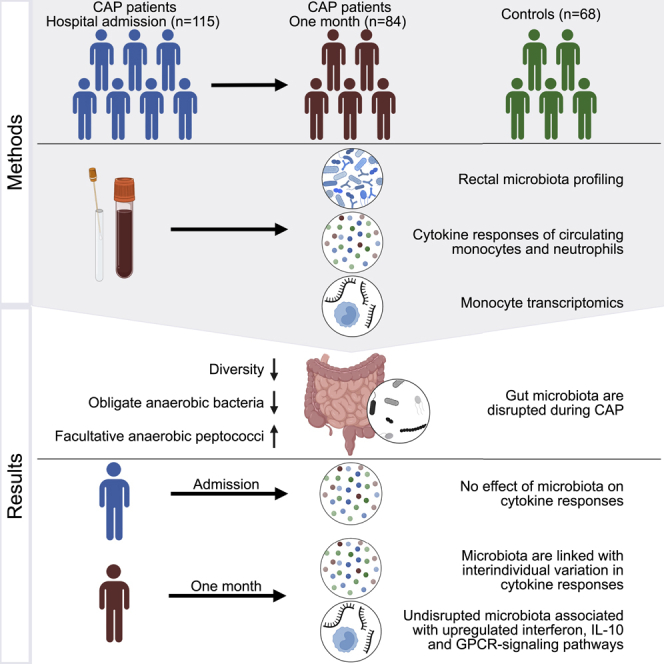

## Introduction

Pneumonia is the leading cause of infectious disease mortality worldwide, responsible for an estimated 1.5 million hospitalizations annually in the United States (GBD 2019 Diseases and Injuries Collaborators, 2020; Ramirez et al., 2017). Approximately one in three patients with community-acquired pneumonia (CAP) die within one year of being hospitalized and one in five patients are readmitted to the hospital within 30 days (Ramirez et al., 2017; Prescott et al., 2014). While our knowledge on potential mechanisms contributing to the high risk of rehospitalization and recurrent infections is limited, it has been hypothesized that patients recovering from respiratory infections display a relatively immunocompromised state in the months following hospital discharge (Torres et al., 2021; van der Poll et al., 2017). In support of these observations, preclinical data showed that impairment of circulating monocyte and neutrophil functions may increase the susceptibility to pneumonia and secondary bacterial infections (Puchta et al., 2016; Liu et al., 2017; McNamee and Harmsen, 2006).

In recent years, our group and others have demonstrated that intestinal microorganisms play a protective role against bacterial and viral pneumonia in murine models, by enhancing the function of innate immune cells (Schuijt et al., 2016; Abt et al., 2012; Deshmukh et al., 2014). For example, circulating monocytes of microbiota-depleted mice become permissive for alphavirus infection through diminished type-I interferon responses (Winkler et al., 2020). In addition, disruption of the intestinal microbiota impaired neutrophil function in neonatal mice, which increased the susceptibility to sepsis caused by *Klebsiella pneumoniae* and *Escherichia coli* (Deshmukh et al., 2014). While the underlying mechanisms that link the intestinal microbiota to systemic immune responses are still being unraveled, it has been demonstrated that microbiota-derived short-chain fatty acids (SCFAs) play an important immunomodulatory role. For example, butyrate drives monocyte-to-macrophage differentiation in mice through inhibition of histone deacetylase (HDAC) 3 via G-protein-coupled receptor (GPCR) signaling, resulting in enhanced antimicrobial activity (Schulthess et al., 2019). Moreover, SCFAs are capable of dampening persistent inflammation in experimental pneumonia through HDAC- and GPCR-mediated pathways as well (Chakraborty et al., 2017; Vinolo et al., 2011).

Recent studies in humans have shown similar associations to those observed in murine models. For example, the cytokine production capacity of macrophages and peripheral blood mononuclear cells of healthy adult men was shown to be partially modulated by the gut microbiota (Schirmer et al., 2016). In addition, high relative abundances of butyrate-producing gut bacteria are independently associated with a reduced risk of developing lower respiratory tract infections in patients receiving allogeneic stem cell transplants (Haak et al., 2018).

While the immunodulatory effects of the intestinal microbiome seem to overlap in mice and humans, animal models might overstate the impact of disrupted gut microbiota on innate immune responses and disease susceptibility (Walter et al., 2020), and this immunomodulatory role is yet to be confirmed in humans with CAP. We hypothesized that disruptions of the rectal microbiota composition are related to an impaired cytokine production capacity of circulating monocytes and neutrophils—both during and following CAP. To this end, we test the association of microbiota profiles in patients with CAP, both on hospital admission and one month following hospitalization, to *ex vivo* cytokine production capacity of circulating monocytes and neutrophils. In addition, we quantified the extent of the variation in cytokine measurements attributed to gut microbiota composition. Moreover, we sought to test associations between potential immunomodulatory bacteria and cytokine production, and analyzed gene expression profiles in circulating monocytes.

## Results

### Study population

We performed a prospective multicenter longitudinal observational study and obtained paired rectal swabs and blood samples from 115 patients admitted for CAP, and from the same patients (n = 84) one month after hospital admission (mean 33.6 ± 7.9 days) between October 2016 and July 2018. Sixty-eight participants, matched for age and sex, without acute infection were included as controls. Patients exposed to systemic antibiotics within 48 h prior to hospital admission were excluded. A CONSORT flowchart of patient inclusion and follow-up is depicted in Figure S1. Of note, clinical characteristics and microbiota composition of 64 of 115 patients with CAP included in the present study (56%) have been described previously by our group (Kullberg et al., 2021).

Patients with CAP and controls did not differ in age, dietary habits, most comorbidities, and antibiotic exposure prior to sampling (Table 1). However, patients with CAP had a lower body mass index (p = 0.01), a higher prevalence of chronic obstructive pulmonary disease (COPD) and an immunosuppressed state compared to controls (p < 0.01; Table 1). Causative pathogens were identified in 60 patients (52.2%); 18 patients (15.7%) had *Streptococcus pneumoniae* infections, 11 patients (9.6%) were infected with *Haemophilus influenzae*, and five had *Staphylococcus aureus* infections (4.3%). Respiratory viruses were diagnosed in 29 patients (25.2%), of whom 12 (10.4%) were attributed to influenza A or influenza B virus (Table S1).

**Table 1:** Clinical characteristics of the study population

	CAP (n = 115)	Controls (n = 68)	p value
**Demographics**			

Age, y, median (IQR)	69 (60–78)	70 (64–75)	0.42
Male sex, n (%)	64 (55.7)	40 (58.8)	0.79
Caucasian ethnicity, n (%)	84 (73.7)	59 (88.1)	0.29
Body Mass Index, median (IQR)	24.8 (21.3–28.1)	26.3 (24.7–28.9)	0.01
Never smoked, n(%)	36 (31.6)	28 (41.2)	0.42
Non-vegetarian diet, n (%)	105 (92.9)	67 (98.5)	0.18
Prior antibiotics^a^, n (%)	16 (13.9)	3 (4.4)	0.07
Influenza vaccination, n (%)	67 (59.3)	23 (33.8)	<0.01

**Chronic comorbidity, n (%)**			

COPD	35 (30.4)	5 (7.4)	<0.01
Cardiovascular disease	87 (75.7)	42 (61.8)	0.07
Diabetes	32 (27.8)	9 (13.2)	0.04
Malignancy	40 (34.8)	15 (22.1)	0.10
Immunosuppressed state^b^	30 (26.1)	5 (7.4)	<0.01
Gastrointestinal disease	18 (15.7)	4 (5.9)	0.08
Chronic renal disease	14 (12.2)	3 (4.4)	0.14

**Severity of disease on admission**			

PSI, median (IQR)	4 (3–4)		
qSOFA, median (IQR)	1 (0–1)		

**Outcome**			

Intensive Care Unit admission, n (%)	9 (7.9)		
Length of hospital stay, days, median (IQR)	4 (3–8)		
28-day mortality	5 (4.5)		

CAP, community-acquired pneumonia; IQR, interquartile range; COPD, chronic obstructive pulmonary disease; PSI, pneumonia severity index; qSOFA, quick Sequential Organ Failure Assessment.

a Exposure to oral or systemic antibiotics between 90 days and 48 h prior to admission.

b Immunosuppressed state was defined as clinically suspected or proven immunodeficiency, the use of immunosuppressive therapy or immunomodulating medication in the past 3 months, including chemotherapy or the use of more than 10mg prednisone or equivalent each day for the past 3 months.

### Alterations in rectal microbiota composition and diversity during and following CAP

**Figure 1:**
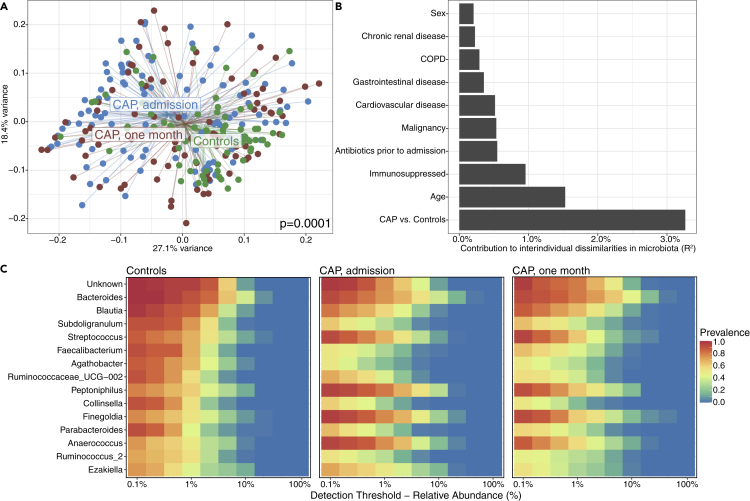
Alterations in rectal microbiota composition and diversity during and following CAP (A) Differences in intestinal microbiota β-diversity with weighted Unifrac distance between patients with CAP at admission (n = 115), after one month (n = 84) and controls (n = 68). Significance of differences in community composition are determined using permutational multivariate ANOVA. (B) The presence of CAP (both at hospital admission and one month following hospitalization) was the strongest determinant of interindividual dissimilarities (R^2^) in rectal microbiota composition when compared to other potential factors determining microbiota composition, determined by permutational multivariate analyses of variance with the weighted Unifrac distance. (C) Controls had high prevalence and relative abundance of *Bacteroides* and members of the Ruminococcaceae and Lachnospiraceae families Ruminococcaceae (e.g. *Blautia, Faecalibacterium*, and *Agathobacter*) while rectal microbiota of patients with CAP were dominated by facultative anaerobic peptococci (e.g. *Finegoldia*, *Peptoniphilus*, and *Anaerococcus*). In the heatmap, the color of each cell shows the percentage of samples (prevalence) in which a specific bacterial genus (y axis) is present at different relative abundances (x axis).

First, we asked whether the composition of rectal microbiota, profiled by sequencing the V3-V4 region of the 16S rRNA gene (STAR methods) (Haak et al., 2022), of patients with CAP was disrupted as compared to controls. In line with our previous findings on a subset of patients included in the present study (Kullberg et al., 2021), rectal bacteria of patients with CAP differed from controls. Although overlap in community composition was observed based on principal coordinate analysis (β-diversity assessed by weighted and unweighted UniFrac), there was a detectable and statistically significant difference between gut microbial profiles of patients with CAP and controls (p = 0.0001; Figures 1A and S2). In addition, the rectal microbiota of patients with CAP displayed lower community diversity when measured by both the Shannon diversity index and Observed taxa richness (p < 0.0001; Figure S2).

The presence of CAP (both at hospital admission and one month following hospitalization) was the strongest determinant of interindividual dissimilarities in rectal microbiota composition when compared to other potential disruptive factors, such as age, sex, comorbidities, and antibiotic exposure in the 48 h—90 days prior to study inclusion. Specifically, CAP explained 3% of the interindividual dissimilarities in microbiota composition (R^2^ = 0.03), whereas prior antibiotic exposure (defined as exposure to antibiotics between 48 h and 90 days prior to hospital admission; patients exposed to antibiotics within 48 h prior to admission were excluded), presence of COPD and an immunosuppressed state explained 0.55%, 0.29%, and 0.96%, respectively (Figure 1B). Notably, when we controlled for potential confounders (age, sex, comorbidities, and prior antibiotic exposure), the association between CAP and rectal bacterial community composition remained significant (p = 0.0001).

Microbiota of patients with CAP were enriched for several facultative anaerobic genera, such as *Streptococcus*, *Peptoniphilus*, *Finegoldia,* and *Anaerococcus*, and a decreased abundance of obligately anaerobic genera pertaining to the families Lachnospiraceae and Ruminococcaceae (e.g. *Blautia, Faecalibacterium*, and *Agathobacter*) (Figure 1C). These data indicate that rectal microbiota composition and diversity were altered at hospital admission for CAP and after one month, when compared to controls.

### Unsupervised clustering of microbiota profiles during and following CAP

Considering the substantial overlap in rectal microbiota composition between patients with CAP and controls, we examined samples of patients with CAP during and following hospitalization for distinct clusters (i.e. subgroups of samples with similar composition) of microbiota composition using unsupervised Dirichlet multinomial mixture (DMM) models (Holmes et al., 2012).

**Figure 2:**
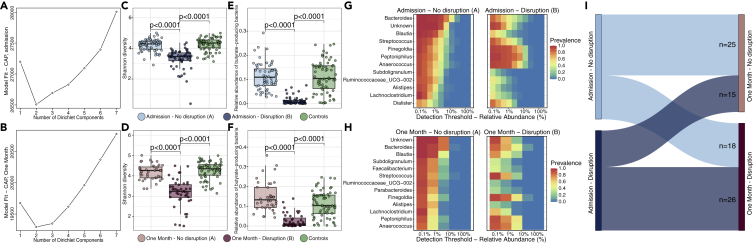
Unsupervised clustering of microbiota profiles during and following CAP Laplace approximation of model fitting showed that two clusters offered the best fit of patients at admission (A) and after one month (B): one with disrupted microbiota (n = 55 at admission; n = 44 after one month) and the other with undisrupted microbiota (n = 60 at admission; n = 40 after one month). Cluster B (“disruption”) had significantly decreased alpha diversity compared to cluster A (“no disruption”) and controls, both at admission (C) and after one month (D), as well as lower relative abundances of butyrate-producing bacteria in the disrupted cluster at admission (E) and after one month (F). The undisrupted cluster had high prevalence and relative abundance of *Bacteroides* and members of the Ruminococcaceae and Lachnospiraceae families (G), while the disrupted cluster was dominated by facultative anaerobic peptococci (e.g. *Finegoldia*, *Peptoniphilus*, and *Anaerococcus*) (H). Sankey diagram showing the number of patients (represented by the size of the links) that remained within the same cluster between the two timepoints, or shifted between microbiota clusters (e.g. from the undisrupted cluster at admission to the disrupted cluster one month thereafter) (I). In the boxplots, the central rectangle spans the first quartile to the third quartile (the interquartile range or IQR), the central line inside the rectangle shows the median, and whiskers above and below the box. Given the non-parametric nature of the data, p values were calculated using the Wilcoxon rank-sum test. In the heatmap, the color of each cell shows the percentage of samples (prevalence) in which a specific bacterial genus (y axis) is present at different relative abundances (x axis).

At both timepoints, Laplace approximation of model fitting showed that two clusters offered the best fit (Figures 2A and 2B), which were robust in 10-fold re-clustering (STAR methods). Both at admission and after one month, one cluster (cluster A; n = 60 at admission; n = 40 after one month) was comparable to controls in terms of community composition, alpha diversity, and the relative abundance of butyrate-producing bacteria, whereas the other cluster (cluster B; n = 55 at admission; n = 44 after one month) had significantly decreased alpha diversity and lower relative abundances of butyrate-producing bacteria (Figures 2C–2F and S3). At both time points, cluster A was characterized by high relative abundance and prevalence of *Bacteroides* and members of the Ruminococcaceae and Lachnospiraceae families, while cluster B appeared to be dominated by facultative anaerobic peptococci (e.g. *Finegoldia*, *Peptoniphilus*, and *Anaerococcus*) (Figures 2G and 2H). These differences in relative abundance were confirmed by DESeq2 analyses (STAR methods) (Love et al., 2014). We identified 94 differentially abundant genera between clusters at admission, and 81 genera after one month (multiple testing adjusted p < 0.05 and ≥2 log2 fold-change; Figure S3). Hence, our unsupervised clustering approach identified two clusters of patients with CAP at both timepoints, one cluster (cluster A) with an undisrupted microbiota profile (i.e. comparable to controls), and one other cluster (cluster B) pertaining to patients with a disrupted microbiota profile.

Profiles were not static over both time points, yet the majority of patients with CAP pertaining to cluster B at admission remained within this disrupted cluster one month later (26 of 44 patients, 59%; Figure 2I). Despite the observed differences in microbial communities, there were no significant differences in comorbidities, causative pathogens, outcomes, or antimicrobial exposure (prior to hospital admission, total duration of antibiotic treatment, nor the types of antibiotics) between disrupted and undisrupted profiles (Figure S3; Table S2).

### Relating rectal microbiome composition to cytokine responses during and following CAP hospitalization

We next used complementary analyses to determine whether rectal microbiota were associated with innate immune cell-specific cytokine production capacity and neutrophil degranulation products. To this end, CD14^+^ monocytes and polymorphonuclear leukocytes (PMNs) were obtained from patients with CAP at both timepoints and stimulated *ex vivo* for 24 h (monocytes) or 2 h (PMNs) with lipopolysaccharide (LPS) or heat-killed *K. pneumoniae*. Following stimulation, we measured a wide array of monocyte-derived cytokines and neutrophil-derived cytokines and degranulation products by multiplex assay (STAR methods and previously reported by our group (Brands et al., 2021)). Of note, we observed no differences in leukocyte, lymphocyte, or neutrophil counts between clusters (Table S2).

**Figure 3:**
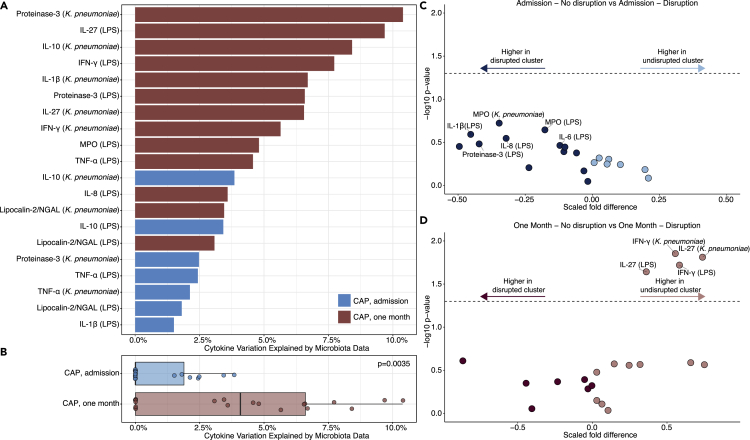
Relating gut microbiota to cytokine responses during and following CAP hospitalization (A) Rectal microbiota accounted for a maximum of 10.4% of interindividual variation in cytokine production capacity of CD14^+^ monocytes and polymorphonuclear cells upon *ex vivo* stimulation with LPS or heat-killed *K. pneumoniae*. (B) Considerably less variation in cytokine responses was explained by the microbiota at hospital admission for CAP compared to at one month following hospitalization. Microbiota are represented by the first four principal coordinates (PCoA with weighted Unifrac distance) that explain ∼65% of the variance, to avoid overestimation due to species-species correlations. The cytokine variance explained by these principal coordinates was estimated through permutation ANOVA by summing over the significant contributions (p < 0.2). In the boxplot, dots represent the percentage of cytokine variation explained by microbiota data for each cytokine response (also shown in panel A), the central rectangle spans the first quartile to the third quartile (the interquartile range or IQR), the central line inside the rectangle shows the median, and whiskers above and below the box. (C) No significant differences (p > 0.05) in cytokine responses between clusters A (n = 55; undisrupted) and B (n = 60; disrupted) at hospital admission. (D) One month following hospitalization, monocytes of patients with undisrupted microbiota profiles (n = 40) produced higher levels of IFN-ɣ and IL-27 compared to patients with disrupted microbiota profiles (n = 44). Given the non-parametric nature of the data, p values were calculated using the Wilcoxon rank-sum test.

First, we quantified how much of the variation in cytokine measurements and degranulation products could be attributed to the rectal microbiota, as described for healthy humans (Schirmer et al., 2016). We represented the microbiota through the first four principal coordinates (PCoA analysis with weighted UniFrac distance) which accounted for ∼65% of the variability in the microbial composition of the samples (Schirmer et al., 2016). The largest percentage of variation in cytokine responses was explained for proteinase-3 (10.4%), IL (interleukin)-27 (9.7%), and IL-10 (8.4%) at one month after CAP (Figure 3A). On hospital admission, the rectal microbiota composition accounted for a maximum of 3.9% of the variation in cytokine responses (for IL-10), compared to a maximum of 10.4% of variability in cytokines and degranulation products one month following hospitalization. Considerably less variation in cytokine responses and degranulation products was explained by the microbiota in the acute phase of CAP (median 0% [IQR 0.0%–1.9%]) than at one month following hospitalization (median 4.1% [IQR 0.0%–6.6%], p = 0.0035; Figure 3B). Similarly, using univariable analysis, no significant differences in cytokine producing capacity and degranulation products were observed between patients with undisrupted (cluster A) and disrupted microbiota profiles (cluster B) on hospital admission (Figure 3C). Whereas after one month, monocytes of patients with undisrupted microbiota (cluster A) produced higher levels of interferon gamma (IFN-ɣ) and IL-27 (in response to both LPS and *K. pneumoniae*) as compared to patients with disrupted microbiota profiles (cluster B; Figure 3D). Controlling for differences in age and sex between clusters in multivariable analyses did not impact these findings.

In addition, we measured a broad range of cytokines and degranulation products in the blood serum of patients with CAP. In line with our findings for the cytokine responses and degranulation products of stimulated monocytes and neutrophils, the largest percentage of variation in serum cytokine responses was explained for cytokines at one month following hospitalization (CRP, IL-6, IL-1RA; Figure S4). While at hospital admission, serum cytokines and degranulation products did not differ between patients with undisrupted and disrupted microbiota profiles; at one month following hospitalization, serum IL-27 was higher in patients with undisrupted microbiota profiles (Figure S4).

Next, considering the potentially immunomodulatory role of the microbiota-derived metabolite butyrate (Schulthess et al., 2019; Chakraborty et al., 2017; Haak et al., 2018), we analyzed associations between the relative abundance of known butyrate-producing bacteria (as listed in Table S3) and cytokine responses (as measured upon *ex vivo* stimulation of monocytes and neutrophils with LPS or heat-killed *K. pneumoniae*) using Spearman correlations. Similar to the comparison between disrupted and undisrupted microbiota clusters at hospital admission, we observed no significant associations between the relative abundance of butyrate-producing bacteria and cytokine responses or degranulation products at this timepoint (Figure S5). However, in patients one month following hospitalization, the abundance of butyrate-producers was correlated with IFN-ɣ (in response to LPS) and IL-27 (p < 0.05; Figure S5).

Together, these data indicate that there is no strong relation between rectal microbiota composition and both the cytokine-producing capacity and release of degranulation products of circulating monocytes or neutrophils at hospital admission for CAP. In contrast, rectal bacterial communities—specifically the abundances of butyrate-producing bacteria—are related to monocyte and neutrophil function one month following hospitalization for CAP.

### Disrupted microbiota profiles are associated with altered monocyte gene sets following CAP hospitalization

Given the observations that monocytes of patients with CAP with disrupted microbiota profiles display an altered production capacity of IL-27 and IFN-ɣ one month following hospital admission, we aimed to determine if monocyte gene expression was altered between the two clusters at that time point. We employed genome-wide transcriptional RNA profiling on monocytes isolated from 53 patients within this cohort without malignant hematological disease, exposure to chemotherapy, systemic corticosteroids, and/or other immunosuppressive drugs (n = 27 with undisrupted microbiota; n = 26 with disrupted microbiota).

**Figure 4:**
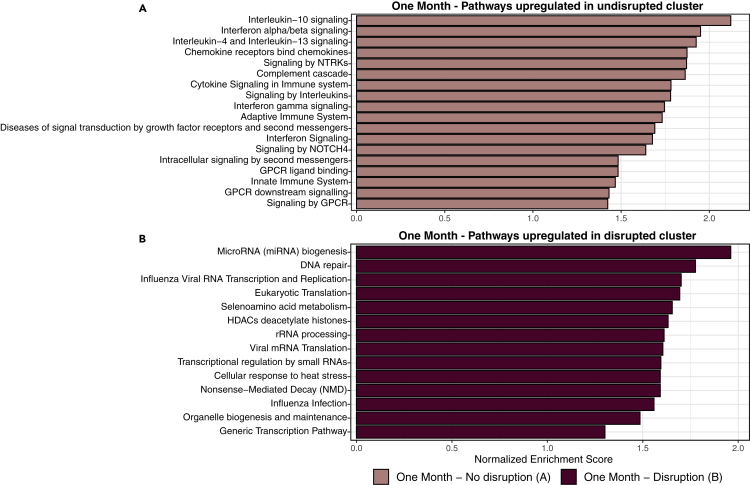
Disrupted microbiota profiles are associated with altered monocyte gene expression pathways following CAP hospitalization Functional gene set enrichment analysis pathway analysis identified several immune response pathways which were significantly enriched in monocytes of patients with undisrupted microbiota profiles (n = 27; top) and pathways enriched in monocytes of patients with disrupted microbiota profiles (n = 26; bottom). To increase interpretability, collapsed functional pathways with Benjamini-Hochberg adjusted p values <0.05 are shown. All significantly altered pathways are depicted in Figure S6.

Initial individual gene expression analysis comparing monocytes of patients with disrupted to those with undisrupted microbiota profiles yielded no significantly enriched transcripts (Figure S6). However, applying gene set enrichment analysis (GSEA), which takes into account differences observed at all genes, yielded several key immune response pathways significantly upregulated in monocytes isolated from patients with undisrupted microbiota profiles compared to monocytes of patients from the disrupted cluster (Figures 4 and S6). In line with the cytokine production assays one month following admission, monocytes of patients with undisrupted microbiota profiles displayed, among others, upregulated IL-10, IFN, and IL4/-13 signaling pathways. In contrast, monocytes of patients with disrupted microbiota profiles displayed upregulation of pathways dedicated to viral replication and proliferation. In addition, patients with an undisrupted rectal microbiota composition had increased expression of GPCR ligand-binding pathways, whereas patients with disrupted microbiota—suggestive of lower relative abundances of butyrate-producing bacteria—had upregulated HDAC pathways. These findings are in line with preclinical studies linking SCFAs, in particular butyrate, to the inhibition HDAC activity via GPCR signaling, resulting in enhanced antimicrobial activity (Schulthess et al., 2019; Chakraborty et al., 2017; Chang et al., 2014).

Taken together, these results suggest that rectal microbiota profiles are associated with altered gene expression pathways of monocytes following CAP hospitalization.

## Discussion

In this prospective longitudinal observational case-control study, we analyzed the relation between the composition of the rectal microbiota and cytokine production and degranulation products of monocytes and neutrophils during CAP. Rectal microbiota composition accounted for no variation in cytokine responses at hospital admission for CAP, while one month following hospitalization rectal microbiota composition was associated with interindividual variation in cytokine responses. Our results suggest a limited role of gut microbiota in modulating circulating innate immune cell function in the acute phase of pneumonia, but may provide insights into potential mechanisms contributing to recurrent infections and rehospitalizations following hospital discharge.

We demonstrate that the composition of the gut microbiota of newly admitted patients with CAP was already altered compared to non-infected controls. These alterations could be a consequence of the disease itself—as murine studies showed that respiratory infection-induced anorexia lead to depletion of SCFA-producing bacteria and enrichment of Proteobacteria (Lankelma et al., 2017a; Sencio et al., 2020). In turn, gut microbiota alterations could predispose patients to the development of CAP, as it has been hypothesized that disrupted microbiota could increase the susceptibility to infections from the outset (Haak et al., 2018; Prescott et al., 2015).

The presence of CAP was the strongest determinant of interindividual dissimilarities in rectal microbiota composition when compared to other potential factors determining microbiota composition and explained 3% of interindividual differences, which is in line with reported effect sizes of clinically relevant variables (e.g. lifestyle and diet) in recent large cohort studies (Falony et al., 2016; Deschasaux et al., 2018). However, we observed considerable overlap in microbiota composition between patients with CAP and control and therefore employed unsupervised clustering to detect distinct metacommunities using unsupervised DMM models. We found two robust clusters of patients with CAP at both timepoints: one cluster representing an undisrupted microbiota profile, which was comparable to healthy controls, and another cluster with disrupted microbiota. The potential sources of such interpatient heterogeneity in gut microbiota remain unclear as comorbidities, severity of disease, exposure to antimicrobial therapy, and underlying causative pathogens were comparable across clusters. Other known putative confounders, like host genetics (Bonder et al., 2016) and altered food or fiber intake over the course of illness (Sencio et al., 2020), were not included in this analysis and may warrant further exploration in future studies.

Rectal microbiota explained up to 10.4% of variability in cytokine responses and degranulation products of stimulated monocytes and neutrophils, which is comparable to earlier estimations in healthy humans and less than other factors, such as host genetics (explaining 25%–50% of the variability in some cytokine responses) (Schirmer et al., 2016; Li et al., 2016). Despite of the stark interindividual differences in gut microbiota composition at hospital admission, the microbiota accounted for only up to 3.9% of the variation in cytokine responses at this timepoint. For two-thirds of the cytokines during the acute phase of CAP, rectal microbiota did not contribute significantly to interindividual variation. Moreover, we observed no differences in the cytokine-producing capacity of monocytes and neutrophils or serum cytokines between clusters at hospital admission, nor did we find differences in numbers of white blood cells in the circulation. We reason that the immunomodulatory crosstalk between intestinal microbes and innate immune responses, as seen in mice and healthy humans (Schuijt et al., 2016; Schirmer et al., 2016), may be either disrupted or overwhelmed in the acute phase of infection, suggesting no important role for gut microbiota in modulating systemic innate cytokine responses in the acute phase of CAP.

One month following hospitalization, rectal microbiota accounted for more variation in cytokines and degranulation products (e.g. in Proteinase-3 and IL-10) and we observed that rectal microbiota disruptions—specifically the abundances of butyrate-producing bacteria—were coupled with altered production of IL-27 and IFN-ɣ. Proteinase-3 is a neutrophil-derived serine protease that can activate pro-IL-1β into its bioactive form, implicated in various inflammatory processes, and an important mediator of the host response to microbial infections (Pham, 2006; Joosten et al., 2009). Although neutrophil elastase—a structurally related serine protease—reduced the abundance of Ruminococcaceae and Lachnospiraceae in the gut of mice (Gill et al., 2012), this is the first study suggesting that systemic neutrophil-derived serine proteases are associated with variations in rectal microbiota composition. IL-10 and IFN-ɣ have important regulatory roles in the host immune response and are involved in sepsis-induced immunosuppression. For example, IL-10 contributes to impairment of the pulmonary antibacterial host response following sepsis (Steinhauser et al., 1999). In addition, a pilot study in human volunteers displayed that administration of IFN-ɣ partially reversed immunoparalysis thereby potentially reducing the incidence of secondary infections (Leentjens et al., 2012).

The link between gut microbiota and the cytokine-producing capacity of monocytes was confirmed by comparing transcriptomes of monocytes between clusters, where various cytokine signaling pathways (e.g. IL-10, IFN, and IL-4/IL-13) were upregulated. In addition to these differences in immune response pathways, GPCR and HDAC signaling were differentially expressed between patients with disrupted and undisrupted gut microbiota. HDAC inhibition by SCFAs, which can signal via GPCRs, has been shown in mice to induce production of antimicrobial peptides and support antibacterial activity (Schulthess et al., 2019). Although our findings validate the existence of this mechanism in humans, whether the moderate differences in cytokine production impact the risk of secondary infections following CAP remains unclear.

An important takeaway of this study is that while differences in microbiota composition were stark between groups, the actual association between these differences and cytokine responses or transcriptional profiles was absent or modest. These findings are in line with earlier findings in healthy adults that alterations of the microbiome do not explain more than 10% of the total variance of systemic immune responses (Schirmer et al., 2016) and gut microbiota disruptions (by broad-spectrum antibiotics) do not affect systemic innate immune responses during endotoxemia (Lankelma et al., 2017b).

### Conclusions

In conclusion, we demonstrate that rectal microbiota composition explained no variation in systemic cytokine responses and degranulation products during the acute phase of CAP, despite profoundly distorted gut microbiota. However, alterations in the composition of the rectal microbiota were related to interindividual variation in cytokine responses following recovery from CAP. These data weaken the hypothesis of a relationship between rectal microbiota profiles and circulating innate immune cell function in the acute phase of respiratory infections, but may provide insights into mechanisms that potentially contribute to recurrent infections and rehospitalizations following CAP recovery.

### Limitations of study

This study has several limitations, such as the limited taxonomic resolution at species level of 16S-based sequencing. Additionally, we did not investigate the role of altered gut microbiota profiles on local innate immune cells, such as alveolar macrophages, which also have important roles in the immune response during pneumonia (Schuijt et al., 2016; Paats et al., 2013). Finally, although a causal effect of gut microbial metabolites on cytokine responses has been shown in animal models (Vinolo et al., 2011; Chang et al., 2014; Ganal et al., 2012), we could not exclude the possibility of a confounding factor explaining the relation between microbiota and cytokine responses due to the observational nature of the current study. For example, reduced food intake during CAP could alter the composition of gut microbiota and influence circulating innate immune cell function (Sencio et al., 2020; Jordan et al., 2019).

## STAR★Methods

### Key resources table

**Table:** 

REAGENT or RESOURCE	SOURCE	IDENTIFIER
**Antibodies**		

Magnetic beads coated with anti-CD14 antibodies	Miltenyi Biotech	Cat#130050201; RRID: AB_2665482

**Bacterial and virus strains**		

LPS from *Escherichia coli*, 100 ng/ml, Ultrapure	Invivogen	Cat#0111:B4
*Klebsiella pneumoniae*	This study	ATCC43816

**Biological samples**		

Rectal swab in Universal Transport Medium	Copan	Cat#349C
Heparin tubes	BD Bioscience	Cat#367874

**Chemicals, peptides, and recombinant proteins**		

Ficoll-Paque PLUS	GE Healthcare Life science	Cat#17144002
Maxwell RSC Whole Blood DNA Kit	Promega	Cat#AS1520
Human Luminex multiplex assay	R&D Systems	Cat#LXSAHM-04
BioPlex 200	BioRad	Cat#X10010027401
AllPrep DNA/RNA/Protein mini kit	Qiagen	Cat#80004
KAPA RNA Hyperprep with RiboErase	Roche	Cat#08098131702

**Deposited data**		

Transcriptional monocyte RNA data	GEO database	GEO database: GSE160329
Microbiome sequence data	European Nucleotide Archive	ENA: PRJEB42265

**Software and algorithms**		

R	R Core Team 2014	Version 4.1.1
Python (Linux/UNIX)	Python	Version 2.7.12

**Other**		

Cell-repellent surface 48-well plate	Greiner Bio-One	Cat#677970
MACS LS columns	Miltenyi Biotech	Cat#130-042-401
Magnetic separator for MACS	Miltenyi Biotech	Cat#130-091-051

### Resource availability

#### Lead contact

Further information and requests for resources and reagents should be directed to and will be fulfilled by the lead contact, Robert Kullberg (r.f.j.kullberg@amsterdamumc.nl).

#### Materials availability

This study did not generate new unique reagents.

### Experimental model and subject details

#### Human cohort

The prospective longitudinal ELDER-BIOME study was performed in the Amsterdam UMC, location Academic Medical Center and the BovenIJ hospital in the Netherlands from October 2016 - June 2017 and October 2017 - June 2018. Consecutive patients older than 18 years admitted to the hospital were screened by trained research physicians. Patients were included if they were admitted with a clinical suspicion of an acute infection of the respiratory tract, defined as the presence of at least one respiratory symptom (new cough or sputum production, chest pain, dyspnea, tachypnea, respiratory failure or abnormal lung examination) and one systemic symptom (documented fever or hypothermia, leukocytosis or leukopenia), combined with an evident new or progressive infiltrate, consolidation, cavitation, or pleural effusion on chest X ray or computed tomography scan (Postma et al., 2015). Patients with the clinical suspicion of an aspiration pneumonia or hospital-associated pneumonia, and patients exposed to oral and/or intravenous antibiotics within 48 hours prior to hospital admission were excluded.

One rectal swab in Universal Transport Medium (Copan, Murrieta, CA) and 50mL of heparin anticoagulated blood were collected within sixteen hours of hospital admission. Subjects without acute infection who presented at the outpatient clinic of the Amsterdam UMC, location AMC, served as controls. Controls were age- and sex-matched on the cohort level. For CAP patients, a second set of samples was collected one month following hospital admission (mean 33.6 ± 7.9 days), either at the residence of the study participant or at the outpatient clinic of the study center. Sex, age and clinical characteristics of study participants are reported in Table 1; sex was self-reported. To further address the influence of sex, we quantified the contribution of sex to interindividual dissimilarities in rectal microbiota composition and included sex as a covariate in multivariable analyses.

For transcriptional RNA profiling of monocytes, patients with previously diagnosed malignant hematological disease, or exposed to chemotherapy, systemic corticosteroids and/or other immunosuppressive drugs were excluded (Brands et al., 2021).

#### Ethics statement

Written informed consent was obtained from all eligible participants, or their legal representatives. The study protocol was approved by the local institutional review boards (reference: NL57847.018.16) and conducted according to the declaration of Helsinki.

### Method details

#### Clinical data collection

Baseline demographics, dietary habits, comorbidities, related medication history, influenza history, as well as exposure to antibiotics in the 90 days prior to admission and throughout follow up was collected for all study participants using questionnaires and from the electronic medical record. For CAP patients, vital parameters and severity scores, such as the quick Sequential Organ Failure Assessment (qSOFA) score (Singer et al., 2016) and Pneumonia Severity Index (PSI) (Fine et al., 1997), were calculated upon hospital admission. Leukocyte counts and differentials were determined using routine diagnostic laboratory methods at the study center (analysis on a Sysmex® XN 9000 analyzer (Sysmex Corporation, Kobe, Japan) by fluorescence flow cytometry in K2EDTA anticoagulated blood) (Schapkaitz and Raburabu, 2018).

#### 16s rRNA gene sequencing

Following collection, rectal swabs were stored at −80°C. Our 16s rRNA gene sequencing protocol and preprocessing pipeline have been earlier described (Haak et al., 2022). After completion of study inclusions, DNA was extracted from the rectal swabs using a repeated bead beating protocol, after which the DNA was purified using the Maxwell RSC Whole Blood DNA Kit (Promega, Madison, WI) (Yu and Morrrison, 2004; Costea et al., 2017). Twenty nanograms of DNA were used for the amplification of the 16S rRNA gene with the V3-V4 341F forward primer and the 805R reverse primer for 25 cycles. The PCR was performed in a total volume of 30 μl containing 1× High Fidelity buffer (Thermo Fisher Scientific, Waltham, MA, USA); 1 μl deoxynucleoside triphosphate (dNTP) mix (10 mM; Promega, Leiden, The Netherlands); 1 U of Phusion green high-fidelity DNA polymerase (Thermo Fisher Scientific, Waltham, MA, USA); 500 nM of the forward 8-nucleotide (nt) sample-specific barcode primer containing the Illumina adapter, pad, and link (341F [5′-CCTACGGGNGGCWGCAG-3′]); 500 nM of the reverse 8-nt sample-specific barcode primer containing the Illumina adapter, pad, and link (805R [5′-GACTACHVGGGTATCTAATCC-3′]); 20 ng/μl of template DNA; and nuclease-free water. The amplification program was as follows: an initial denaturation step at 98°C for 30 s; 25 cycles of denaturation at 98°C for 10 s, annealing at 55°C for 20 s, and elongation at 72°C for 90 s; and an extension step at 72°C for 10 min (Kozich et al., 2013). The size of the PCR products (∼540 bp) was confirmed by gel electrophoresis using 4 μl of the amplification reaction mixture on a 1% (w/v) agarose gel containing ethidium bromide (AppliChem GmbH, Darmstadt, Germany).

PCR products were purified using AMPure XP beads (Beckman Coulter, Brea, CA, USA). The amplicon DNA concentration was measured using the Qubit fluorometric quantitation method (Thermo Fisher Scientific, Waltham, MA, USA), after which the purified products were equimolarly pooled. Purified amplicon pools were 250 bp paired-end sequenced with 2x251 cycles on an Illumina MiSeq platform (GATCBiotech, Constance, Germany) using V3 chemistry, according to manufacturer’s instructions. Performance was evaluated by inclusion of two positive controls consisting of genomic material of 55 different strains.

#### Monocyte and neutrophil isolation

Monocyte isolation and purity verification have been earlier described (Brands et al., 2021). Heparinized blood was diluted (1:1) with phosphate-buffered saline (PBS). Peripheral blood mononuclear cells (PBMCs) and polymorphonuclear leukocytes (PMNs) were obtained by density centrifugation (1700 RPM for 30 min at 21°C, acceleration 1, breaks 0) of diluted blood over Ficoll-Paque PLUS (GE Healthcare Life science, Little Chalfont, UK). For PMNs, erythrocytes were lysed in erythrocyte lysis buffer (Qiagen, Hilden, Germany). PBMCs and PMNs were washed twice with cold PBS supplemented with 0.5% sterile endotoxin-free bovine serum albumin (Divbio Science Europe, Breda, the Netherlands). PMNs were subsequently resuspended in Medium (Roswell Park Memorial Institute medium with 10% sterile fetal calf serum, 200 mM Glutamax, 100 uM Pyruvate and 50 ug/ml gentamycin), while PBMCs were resuspended in MACS buffer (PBS having endotoxin-free 0.5% bovine serum albumin, 2mM ethylenediaminetetraacetic acid (EDTA)), containing magnetic beads coated with anti-CD14 antibodies (Miltenyi Biotec, Bergisch Gladbach, Germany, RRID: AB_2665482), and incubated for 15 min on a roller bank kept at 4°C. CD14^+^ monocytes were purified using a LS MACS column (Miltenyi Biotec) and magnetic separator (Miltenyi Biotec). To determine the purity of PMNs, we incubated PMNs with cell-specific CD66b antibodies and verified purity via flow cytometry (FACS Canto II with FACSDiva Software; BD Biosciences, Heidelberg, Germany). After exclusion of doublets and cell debris, the median proportion of CD66b positive (+) granulocytes within the whole cell fraction (i.e. neutrophil purity) was 94.1% (IQR 86.2-97.4%; data not shown).

#### Cytokine production capacity assays

Directly following isolation, CD14^+^ monocytes and PMNs were adjusted to 2.5 x 10^6^ cells/mL, seeded in 48-well non-repellent plates (Greiner Bio-One, Kremsmünster, Austria; 0.5 x 10^6^ cells/well) and stimulated with lipopolysaccharide (LPS; *Escherichia coli* 0111:B4 Ultrapure, 100 ng/mL, Invivogen, Toulouse, France), or heat-killed (30 minutes at 70°C) *Klebsiella (K.) pneumoniae* (ATCC43816; equivalent of 12.5 x 10^6^ CFU/mL). After 24 hours (monocytes) or 2 hours (PMNs) of stimulation, samples were centrifuged for 8 min at 1400 RPM at 4°C and supernatants were stored at −80°C until further analysis. One technical replicate per sample was run. Following completion of study inclusions and follow-up, concentrations of monocyte-derived cytokines (Tumor Necrosis Factor-α, Interferon-ɣ, Interleukin (IL)-1β, IL-6, IL-10, and IL-27) and neutrophil-derived cytokines (IL-8, myeloperoxidase (MPO), proteinase-3, and Lipocalin-2/neutrophil gelatinase-associated lipocalin (NGAL)) were measured in supernatants of stimulated CD14^+^ monocytes and PMNs respectively, using a Luminex multiplex assay (R&D Systems Inc, Minneapolis, MN) on a BioPlex 200 (BioRad, Hercules, CA), in one run using one batch of assay reagents. In addition, a Luminex multiplex assay was used to measure a wide range of cytokines and degranulation products (CRP, IL-6, IL-10, IL-27, IL-1RA, IL-17, IL-23, Cluster of Differentiation (CD) 163, ferritin, IL-8, MPO, proteinase-3 and Lipocalin-2/NGAL) in the blood serum of CAP patients. Samples that were above the upper limit of quantification were set at the upper detection limit of the assay, while samples below detection limit were set at half of the of the lower detection limit.

#### Monocyte RNA sequencing

A total of 1 × 10^6^ directly harvested CD14^+^ monocytes were stabilized in 350 ul RNAprotect Cell Reagent (Qiagen, Hilden, Germany), after which RNA was isolated using the AllPrep DNA/RNA/Protein Mini Kit according to the manufacturer’s instructions (Qiagen). Sequencing libraries were prepared by means of the KAPA RNA HyperPrep with RiboErase (Roche, Basel, Switzerland) as per manufacturer’s instructions. Libraries were sequenced using the Illumina HiSeq4000 (Illumina, San Diego, CA, USA) to generate single reads (50bp). Sequencing depth was approximately 40 million reads per sample. Sequence read quality was assessed by means of the FastQC method (v0.11.5; http://www.bioinformatics.babraham.ac.uk/projects/fastqc/). Trimmomatic (v 0.36) was used to trim Illumina adapters and poor-quality bases (parameters: leading = 3, trailing = 3, sliding window = 4:15, minimum length = 40).

### Quantification and statistical analysis

Statistical analysis was performed in the R statistical framework (Version 4.1.1, R Core Team 2014. R: A language and environment for statistical computing. R Foundation for Statistical Computing, Vienna, Austria).

#### Microbiota data preprocessing

Following 16s rRNA gene sequencing, sequence reads were analyzed as follows. Read pairs with perfect matching forward and reverse barcodes were assigned to their corresponding samples. Forward and reverse reads were truncated to 240 and 210 bases, respectively, and merged using USEARCH (Edgar, 2010). Merged reads that did not pass the Illumina chastity filter, had an expected error rate higher than 2, or were shorter than 380 bases were filtered. The DADA2 package was used to infer, in parallel, the amplicon sequence variants (ASV), with a minimum abundance of 4 reads (Callahan et al., 2016). Unfiltered reads were than mapped against the collective ASV set to determine the abundances. Taxonomy was assigned using the RDP classifier and SILVA 16S ribosomal database V132 (Wang et al., 2007; Quast et al., 2013). A phylogenetic tree was constructed from the resulting multiple sequence alignment using a generalized time-reversible model with the ‘double-precision’ build of FastTree. The ASV table was integrated with the taxonomy and tree using the phyloseq package (McMurdie and Holmes, 2013). Contaminants were identified using the package decontam, manually scrutinized (frequency method) and subsequently, together with known lab specific contaminants, removed from the dataset (Davis et al., 2018). In total, seven contaminating ASVs were removed.

#### Microbiota data analysis

We calculated alpha diversity (within sample diversity) using the Observed Taxa richness (the sum of unique bacteria in each sample) and the Shannon diversity index (which combines the richness of a sample along with the relative abundance of the present bacteria) with the phyloseq package (McMurdie and Holmes, 2013).

β-diversity (interindividual dissimilarities) was assessed using the weighted and unweighted UniFrac distance (which take the bacterial phylogeny into account), and visualized using principal coordinates analysis with the phyloseq package (McMurdie and Holmes, 2013). PERMANOVA models (vegan package (Oksanen et al., 2017), functions Adonis and Adonis2, 9999 permutations) were used to assess the contribution of different variables (CAP, age, sex, comorbidities and antibiotic exposure in the 48h-90 days before hospital admission) to interindividual dissimilarities in intestinal microbiota composition (β-diversity using the weighted UniFrac distance).

Given the potential role butyrate in the context of CAP, we measured the relative abundance of 17 bacteria that are known to be the most abundant drivers of butyrate production (based on a metagenomic overview which analyzed butyrate-producing pathways on 2387 metagenomic/transcriptomic samples from 15 publicly available data sets (Vital et al., 2017); listed in Table S3) as earlier described by our group (Haak et al., 2018).

To identify differentially abundant genera, we used the ‘DESeq’ function in DESeq2 with Benjamini-Hochberg correction for multiple comparisons (significance threshold α < 0.05 and ≥2 log2 fold-change) (Love et al., 2014). We restricted our DESeq analysis to bacterial genera that were present at greater than 10% of the sample population.

In accordance with recent consensus statements concerning the unsupervised analysis of microbial communities (Costea et al., 2018), we applied unsupervised Dirichlet Multinomial Mixture (DMM) clustering with Laplace approximations to define the optimal number of clusters (i.e. distinct meta-communities) of CAP patients at each timepoint (admission and one month thereafter) (Holmes et al., 2012). For clustering purposes (i.e. dividing sample pools into subgroups with similar composition), DMM models represent a statistically rigorous approach and have been shown to outperform traditional Gaussian multivariate techniques (Holmes et al., 2012; Costea et al., 2018). DMM clusters were derived using count data in a rarefied dataset at the genus level. To examine the robustness of DMM clustering, we randomly divided the patients at admission, and patients one month following hospitalization into ten groups and performed 10-fold DMM clustering, leaving one group out at every occasion (i.e. using 90% of the patients) for both of the timepoints. Thereby, every patient was re-clustered nine times using 90% of the data (the 10th time it was left out), in addition to the original DMM clustering based on all data. Next, we checked concordance of the original clustering against the clustering using 90% of the data. CAP patients at admission belonged to the same clusters in 99.9% of the re-clusterings (once a different cluster of 1035 re-clusterings), and patients at one month in 98.9% (8 times a different cluster of 756 re-clusterings), thus showing the robustness of the DMM clustering.

#### Cytokine data analysis

We used complementary analyses to assess the relation between gut microbiota and cytokine measurements (following stimulation of monocytes and neutrophils or in unstimulated blood serum). First, we quantified how much of the variation in cytokine measurements could be attributed to the gut microbiota, as earlier described for healthy humans (Schirmer et al., 2016). We represented the microbiota through the first 4 principal coordinates (PCoA analysis with weighted UniFrac distance) to avoid overestimation due to species-species correlations. The cytokine variance explained by these principal coordinates was estimated through permutation ANOVA (vegan package (Oksanen et al., 2017), Adonis function, 999 permutations) by summing over the significant contributions (p < 0.2). Next, we compared the cytokine producing capacity between patients with a disrupted and an undisrupted microbiota profile. Data were not normally distributed and are therefore analyzed using a Wilcoxon rank sum test in univariable analyses. ANOVA models were used to control for differences in age and sex between cohorts. Next, we analyzed associations between the cytokine producing capacity of monocytes and neutrophils with the relative abundance of known butyrate-producing bacteria (listed in Table S3) using Spearman correlation coefficient. The two-tailed level of significance was set at p < 0.05.

#### Monocyte RNA analysis

After pre-processing, the remaining high-quality reads were aligned against the human Genome Reference Consortium Build 38 (GRCh38, Ensembl 84) using Hisat2 (2.2.0) (Kim et al., 2015) with default parameters. Count data were generated by means of the HTSeq method (Anders et al., 2015) and differential expression analyzed using the DESeq2 package (Love et al., 2014). Significance was calculated using Benjamini-Hochberg adjusted p-values (α < 0.2). Finally, Gene Set Enrichment Analysis (GSEA) was applied to the Reactome pathway knowledgebase (Yu and He, 2016; Fabregat et al., 2018), to determine the level of enrichment for each pathway.

### Additional resources

This study was part of the ELDER-BIOME project which is registered at clinicaltrials.gov with identifier NCT02928367.

## Data Availability

• Transcriptional RNA data and microbiome sequence data have been deposited at the Gene Expression Omnibus of the National Center for Biotechnology Information: GSE160329 and the European Nucleotide Archive: PRJEB42265, respectively, and are publicly available. Accession numbers are listed in the key resources table. • Original code used for analysis is publicly available at github.com/rfjkullberg/microbiota_cytokine_production. • Any additional information required to reanalyze the data reported in this paper is available from the lead contact upon request.
