# Supplementary material for: Rectal microbiota are coupled with altered cytokine production capacity following community-acquired pneumonia hospitalization
Source: iScience. 2022 Jul 14;25(8):104740. doi: 10.1016/j.isci.2022.104740 (PMC9352523; doi:10.1016/j.isci.2022.104740)
Supplement: Document S1. Figures S1–S6 and Tables S1–S3 [file mmc1.pdf]

iScience, Volume *25*

Supplemental information

Rectal microbiota are coupled with altered cytokine production capacity following community-acquired pneumonia hospitalization

Robert F.J. Kullberg, Xanthe Brands, Augustijn M. Klarenbeek, Joe M. Butler, Natasja A. Otto, Daniël R. Faber, Brendon P. Scicluna, Tom van der Poll, W. Joost Wiersinga, and Bastiaan W. Haak

	n (%)
**Causative pathogen**	
Streptococcus pneumoniae	18 (15.7)
Haemophilus influenzae	11 (9.6)
Influenza A virus	8 (7.0)
Rhinovirus	8 (7.0)
Staphylococcus aureus	5 (4.3)
Coronavirus	4 (3.5)
Influenza B virus	4 (3.5)
Pseudomonas aeruginosa	2 (1.7)
Human MetaPneumoVirus (hMPV)	2 (1.7)
Parainfluenza virus 1-4	2 (1.7)
Pneumocystis jirovecii	2 (1.7)
Aspergillus spp	1 (0.9)
Escherichia coli	1 (0.9)
Other[a]	5 (4.3)
**Type of causative pathogen**	
Bacterial pathogen only	27 (23.5)
Viral pathogen only	18 (15.7)
Fungal pathogen only	1 (0.9)
Bacterial-viral co-detection	9 (7.8)
Viral-fungal co-detection	2 (1.7)
Bacterial-bacterial co-detection	3 (2.6)
No pathogen detected	55 (47.8)

Table S1. Overview of causative pathogens of CAP patients, Related to Table 1
Overview of causative pathogens of CAP patients (n=115) identified by a combination of viral nasal/throat swab polymerase chain reaction, urine antigen tests, microbiological blood and sputum cultures. Cumulative overview of causative pathogens (upper part) and the proportion of bacterial, viral, and mixed cases within the cohort (lower part).

[a] Other pathogens constitute *Rothia dentocariosa*, *Stenotrophomonas maltophilia, Mycobacterium tuberculosis, Moraxella osloensis*, and *Streptococcus salivarius*.

	CAP, admission			CAP, one month		
	No disruption (A; n =60)	Disruption (B; n=55)	p-value	No disruption (A; n=40)	Disruption (B; n=44)	p-value
**Demographics**						
Age, y, median (IQR)	70 (63-78)	67 (50-77)	0.04	69 (60-80)	67 (51-74)	0.02
Male sex, n (%)	32 (53.3)	32 (58.2)	0.74	30 (75.0)	21 (47.7)	0.02
Caucasian ethnicity, n (%)	47 (79.7)	37 (67.3)	0.09	33 (82.5)	31 (72.1)	0.49
Body Mass Index, median (IQR)	25.7 (23.1-27.6)	24.5 (20.7-29.7)	0.35	25.9 (23.1-27.8)	25.4 (21.1-28.1)	0.27
Never smoked, n(%)	16 (27.1)	20 (36.4)	0.44	11 (27.5)	19 (44.2)	0.11
Non-vegetarian diet, n (%)	56 (94.9)	49 (90.7)	0.62	40 (100)	37 (88.1)	0.07
Influenza vaccination, n (%)	41 (69.5)	26 (48.1)	0.06	24 (60.0)	25 (59.5)	0.80
**Chronic comorbidity, n (%)**						
COPD	20 (33.3)	15 (27.3)	0.62	14 (35.0)	10 (22.7)	0.32
Cardiovascular disease	49 (81.7)	38 (69.1)	0.18	32 (80.0)	30 (68.2)	0.33
Diabetes	17 (28.3)	15 (27.3)	1.00	11 (27.5)	9 (20.5)	0.62
Malignancy	21 (35.0)	19 (34.5)	1.00	17 (42.5)	13 (29.5)	0.31
Immunosuppressed[a]	15 (25.0)	15 (27.3)	0.95	11 (27.5)	13 (29.5)	1.00
Gastrointestinal disease	12 (20.0)	6 (10.9)	0.28	4 (10.0)	9 (20.5)	0.31
Chronic renal disease	9 (15.0)	5 (9.1)	0.50	5 (12.5)	5 (11.4)	1.00
**Severity of disease on admission**						
PSI, median (IQR)	4 (3-4)	4 (2-4)	0.09	4 (3-4)	4 (2-4)	0.07
qSOFA, median (IQR)	1 (0-1)	1 (0-1)	0.72	1 (0-1)	1 (0-1)	0.49
**White blood cell counts**						
Leukocytes (cells/microL), median (IQR)	12200 [9850, 15800]	11700 [7800, 17500]	0.34	11600 [7730, 15530]	11700 [8200, 14750]	0.46
Lymphocytes (cells/microL), median (IQR)	1030 [665, 1770]	910 [578, 1310]	0.25	920 [640, 1405]	1000 [735, 1445]	0.32
Neutrophils (cells/microL), median (IQR)	9535 [6777, 13900]	9605 [7112, 12395]	0.87	9000 [6770, 11860]	10040 [7320, 14570]	0.34

	CAP, admission			CAP, one month		
	No disruption (A; n =60)	Disruption (B; n=55)	p-value	No disruption (A; n=40)	Disruption (B; n=44)	p-value
**Causative pathogen**			0.72			0.75
No pathogen identified	30 (50.0)	25 (45.5)		16 (40.0)	24 (54.5)	
Bacterial pathogen	17 (28.3)	13 (23.6)		13 (32.5)	11 (25.0)	
Viral pathogen	9 (15.0)	9 (16.4)		7 (17.5)	5 (11.4)	
Bacterial-viral co-infection	3 (5.0)	6 (10.9)		3 (7.5)	3 (6.8)	
Other[b]	1 (1.7)	2 (3.6)		1 (2.5)	1 (2.3)	
**Antibiotic exposure**						
Antibiotics between 90 days and 48 prior to admission[c], n (%)	8 (13.3)	8 (14.5)	1.00	6 (15.0)	4 (9.1)	0.62
Antibiotic treatment between admission and follow-up, days, median (IQR)	NA	NA	NA	8.0 (6.5-11.5)	8.0 (6.0-16.0)	0.36
Cephalosporin				27 (67.5)	33 (75.0	0.60
Penicillin				31 (77.5)	33 (75.0)	0.99
Fluoroquinolone				17 (42.5)	15 (34.1)	0.57
Metronidazole				0 (0.0)	3 (6.8)	0.27
Other				8 (20.0)	15 (34.1)	0.23
**Outcome & Treatment**						
Time between admission and follow-up, days, median (IQR)	NA	NA	NA	32 (29-38)	33 (30-36)	0.73
Intensive Care Unit admission, n (%)	6 (10.2)	3 (5.5)	0.56	3 (7.5)	3 (7.0)	1.00
Length of hospital stay, days, median (IQR)	4 (3-8)	5 (3-10)	0.29	4 (3-6)	4 (2-7)	0.80
28-day mortality	4 (7.0)	1 (1.8)	0.65	NA	NA	NA

Table S2. Clinical characteristics of patients per cluster, Related to Figure 2

Unsupervised clustering (Dirichlet multinomial mixture models) detected two clusters at both timepoints: one with undisrupted microbiota (cluster A) and the other with disrupted microbiota (cluster B).

Abbreviations: CAP, community-acquired pneumonia; IQR, interquartile range; COPD, chronic obstructive pulmonary disease; PSI, pneumonia severity index; qSOFA, quick Sequential Organ Failure Assessment. [a] Immunosuppressed state was defined as clinically suspected or proven immunodeficiency, the use of immunosuppressive therapy or immunomodulating medication in the past 3 months, including chemotherapy or the use of more than 10mg prednisone or equivalent each day for the past 3 months. [b] Other causative pathogens include mycobacterial and fungal pathogen, and fungal-viral co-infection. [c] Patients exposed to systemic antibiotics within 48 h prior to hospital admission were excluded.

Phylum	Order	Class	Family	Genus
Bacteroidetes	Bacteroidia	Bacteroidales	Marinifilaceae	Butyricimonas
Bacteroidetes	Bacteroidia	Bacteroidales	Marinifilaceae	Odoribacter
Bacteroidetes	Bacteroidia	Bacteroidales	Rikenellaceae	Alistipes
Firmicutes	Clostridia	Clostridiales	Eubacteriales	Eubacterium
Firmicutes	Clostridia	Clostridiales	Lachnospiraceae	Anaerostipes
Firmicutes	Clostridia	Clostridiales	Lachnospiraceae	Butyrivibrio
Firmicutes	Clostridia	Clostridiales	Lachnospiraceae	Coprococcus_2
Firmicutes	Clostridia	Clostridiales	Lachnospiraceae	Coprococcus_3
Firmicutes	Clostridia	Clostridiales	Lachnospiraceae	Roseburia
Firmicutes	Clostridia	Clostridiales	Lachnospiraceae	Shuttleworthia
Firmicutes	Clostridia	Clostridiales	Ruminococcaceae	Butyricicoccus
Firmicutes	Clostridia	Clostridiales	Ruminococcaceae	Faecalibacterium
Firmicutes	Clostridia	Clostridiales	Ruminococcaceae	Flavonifractor
Firmicutes	Clostridia	Clostridiales	Ruminococcaceae	Pseudoflavonifractor
Firmicutes	Clostridia	Clostridiales	Ruminococcaceae	Oscillibacter
Firmicutes	Clostridia	Clostridiales	Ruminococcaceae	Ruminococcus_2
Firmicutes	Clostridia	Clostridiales	Ruminococcaceae	Subdoligranulum

Table S3. Butyrate-producing bacteria selection, Related to STAR Methods
In order to assess the presence of butyrate-producing bacteria in our cohort, we measured the relative abundance of 17 bacteria that are known to be the most abundant drivers of butyrate production.

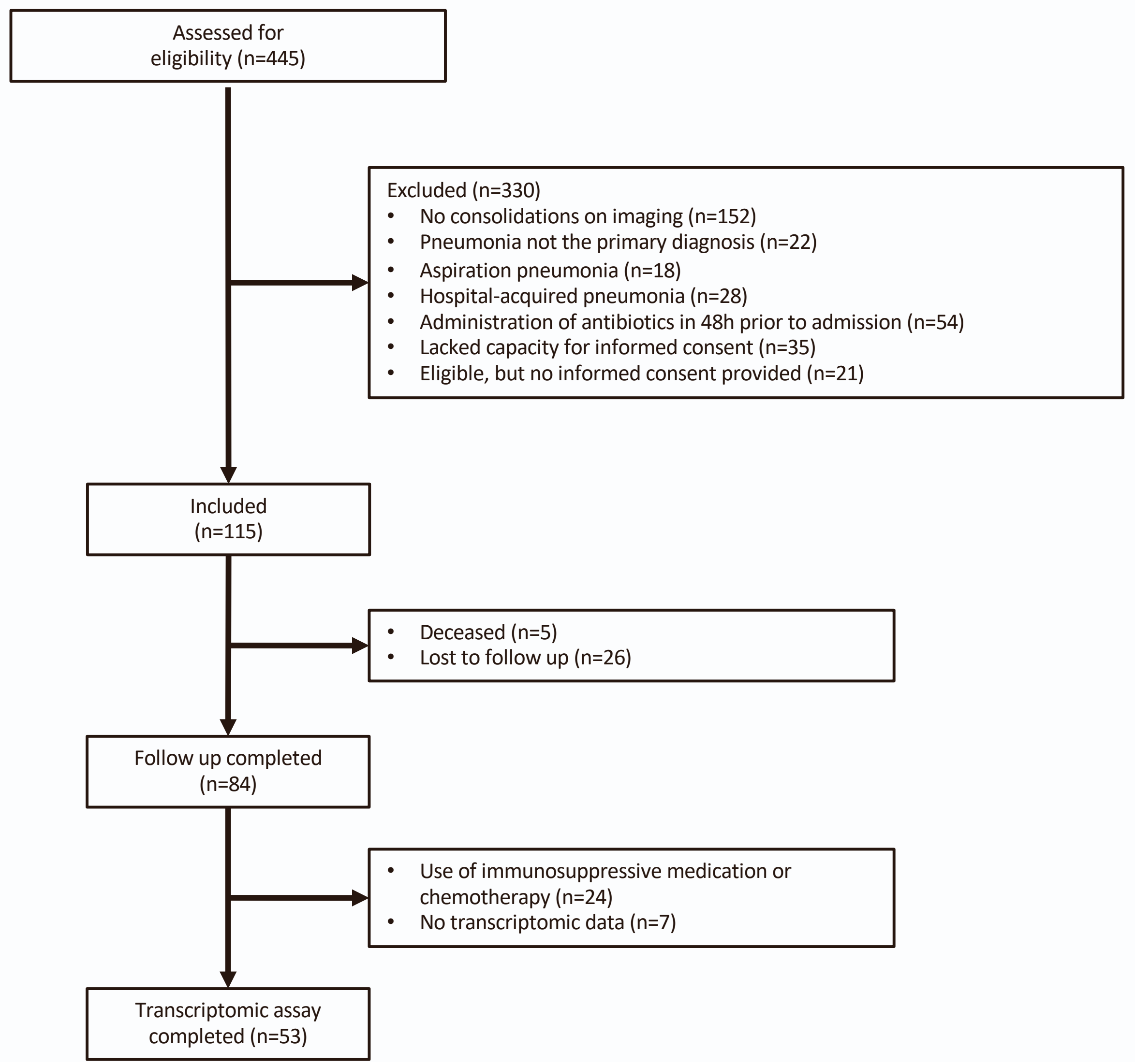

Supplemental Figure 1. CONSORT flow diagram of study participants and follow up, Related to STAR Methods

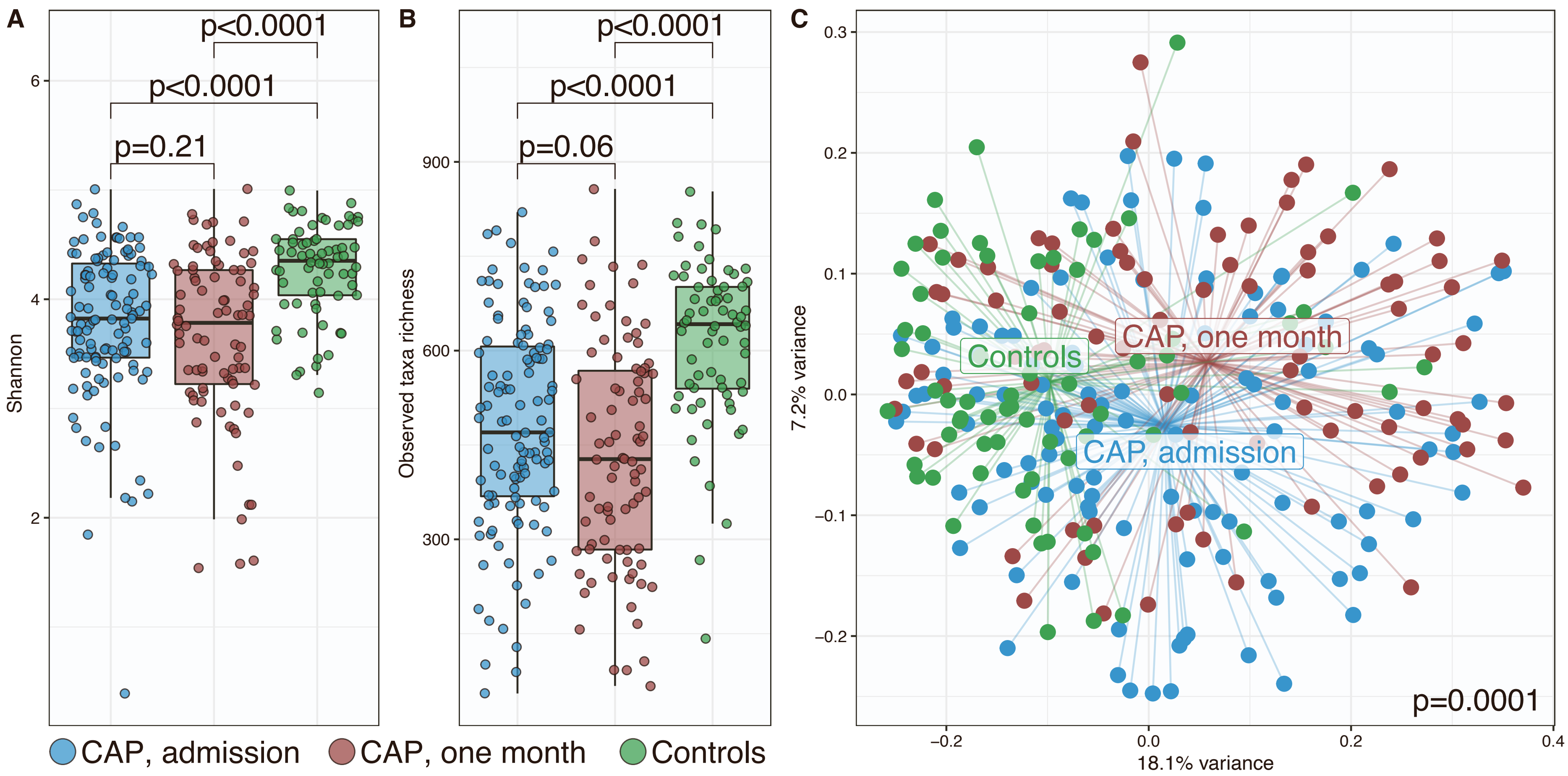

Figure S2. Difference in alpha and beta diversity between CAP patients and controls, Related to Figure 1

Differences in rectal microbiota community diversity (alpha) measured by both the Shannon diversity index (A) and Observed taxa richness (B) between CAP patients at admission (n=115), after one month (n=84) and controls (n=68). Community composition of CAP patients (ß-diversity with unweighted UniFrac distance) differed from controls (C). In the boxplots, the central rectangle spans the first quartile to the third quartile, the central line inside the rectangle shows the median, and whiskers above and below the box. Given the non-parametric nature of the data, p-values were calculated using the Wilcoxon rank sum test.

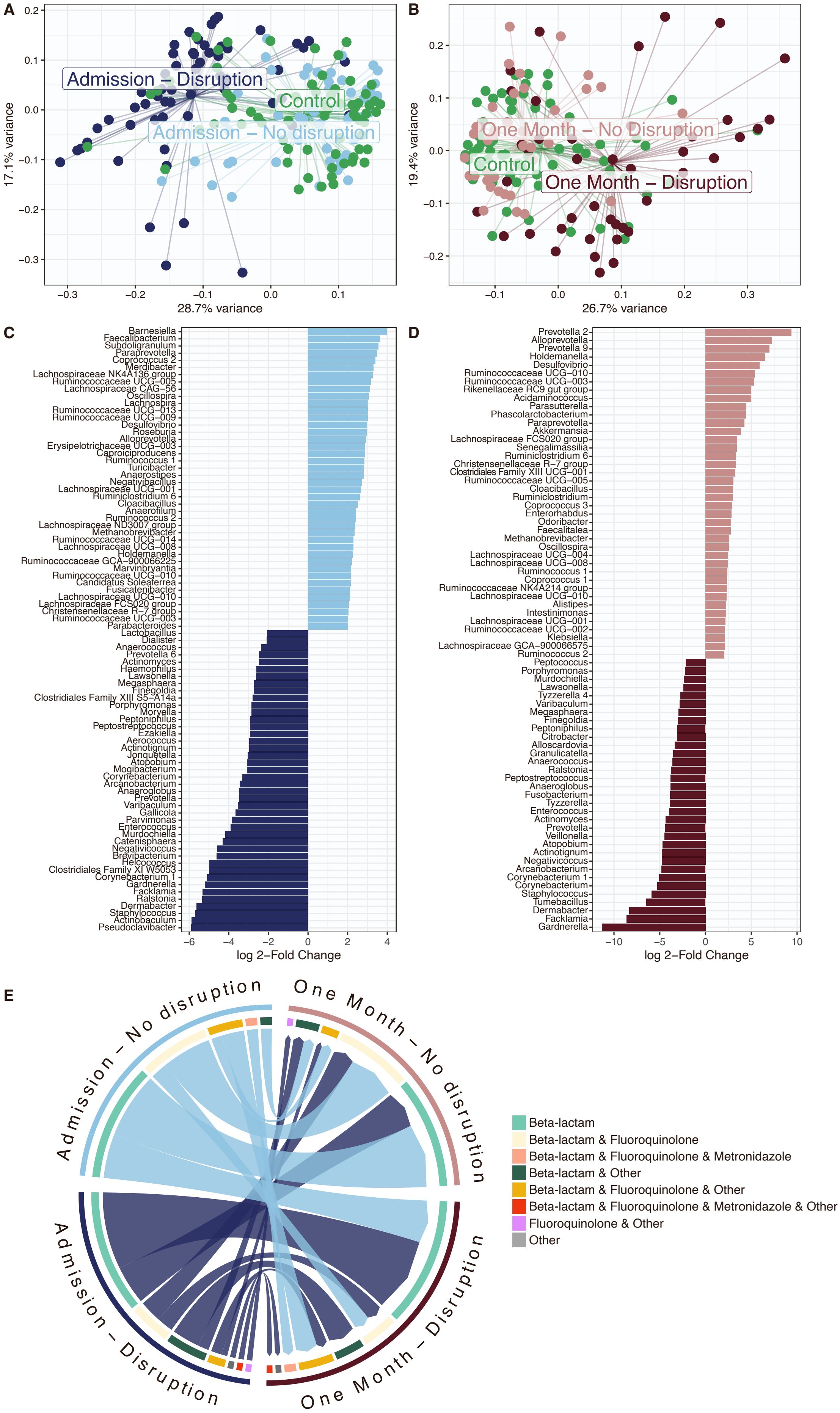
A
Admission – Disruption
Control
Admission – No disruption
17.1% variance
28.7% variance
B
One Month – No Disruption
Control
One Month – Disruption
19.4% variance
26.7% variance
C
Barnesiella
Faecalibacterium
Subdoligranulum
Paraprevotella
Coprococcus 2
Merdibacter
Lachnospiraceae NK4A136 group
Ruminococcaceae UCG−005
Lachnospiraceae CAG−56
Oscillospira
Lachnospira
Ruminococcaceae UCG−013
Ruminococcaceae UCG−009
Desulfovibrio
Roseburia
Alloprevotella
Erysipelotrichaceae UCG−003
Caproiciproducens
Ruminococcus 1
Turicibacter
Anaerostipes
Negativibacillus
Lachnospiraceae UCG−001
Ruminiclostridium 6
Cloacibacillus
Anaerofilum
Ruminococcus 2
Lachnospiraceae ND3007 group
Methanobrevibacter
Ruminococcaceae UCG−014
Lachnospiraceae UCG−008
Holdemanella
Ruminococcaceae GCA−900066225
Marvinbryantia
Ruminococcaceae UCG−010
Candidatus Soleaferrea
Fusicatenibacter
Lachnospiraceae UCG−010
Lachnospiraceae FCS020 group
Christensenellaceae R−7 group
Ruminococcaceae UCG−003
Parabacteroides
Lactobacillus
Dialister
Anaerococcus
Prevotella 6
Actinomyces
Haemophilus
Lawsonella
Megasphaera
Finegoldia
Clostridiales Family XIII S5−A14a
Porphyromonas
Moryella
Peptoniphilus
Peptostreptococcus
Ezakiella
Aerococcus
Actinotignum
Jonquetella
Atopobium
Mogibacterium
Corynebacterium
Arcanobacterium
Anaeroglobus
Prevotella
Varibaculum
Gallicola
Parvimonas
Enterococcus
Murdochiella
Catenisphaera
Negativicoccus
Brevibacterium
Helcococcus
Clostridiales Family XI W5053
Corynebacterium 1
Gardnerella
Facklamia
Ralstonia
Dermabacter
Staphylococcus
Actinobaculum
Pseudoclavibacter
log 2−Fold Change
D
Prevotella 2
Alloprevotella
Prevotella 9
Holdemanella
Desulfovibrio
Ruminococcaceae UCG−010
Ruminococcaceae UCG−003
Rikenellaceae RC9 gut group
Acidaminococcus
Parasutterella
Phascolarctobacterium
Paraprevotella
Akkermansia
Lachnospiraceae FCS020 group
Senegalimassilia
Ruminiclostridium 6
Christensenellaceae R−7 group
Clostridiales Family XIII UCG−001
Ruminococcaceae UCG−005
Cloacibacillus
Ruminiclostridium
Coprococcus 3
Enterorhabdus
Odoribacter
Faecalitalea
Methanobrevibacter
Oscillospira
Lachnospiraceae UCG−004
Lachnospiraceae UCG−008
Ruminococcus 1
Coprococcus 1
Ruminococcaceae NK4A214 group
Lachnospiraceae UCG−010
Alistipes
Intestinimonas
Lachnospiraceae UCG−001
Ruminococcaceae UCG−002
Klebsiella
Lachnospiraceae GCA−900066575
Ruminococcus 2
Peptococcus
Porphyromonas
Murdochiella
Lawsonella
Tyzzerella 4
Varibaculum
Megasphaera
Finegoldia
Peptoniphilus
Citrobacter
Alloscardovia
Granulicatella
Anaerococcus
Ralstonia
Peptostreptococcus
Anaeroglobus
Fusobacterium
Tyzzerella
Enterococcus
Actinomyces
Prevotella
Veillonella
Atopobium
Actinotignum
Negativicoccus
Arcanobacterium
Corynebacterium 1
Corynebacterium
Staphylococcus
Tumebacillus
Dermabacter
Facklamia
Gardnerella
log 2−Fold Change
E
Admission – No disruption
One Month – No disruption
One Month – Disruption
Admission – Disruption
Beta-lactam
Beta-lactam & Fluoroquinolone
Beta-lactam & Fluoroquinolone & Metronidazole
Beta-lactam & Other
Beta-lactam & Fluoroquinolone & Other
Beta-lactam & Fluoroquinolone & Metronidazole & Other
Fluoroquinolone & Other
Other

Figure S3. Unsupervised microbiota clustering results in a disrupted and undisrupted cluster, Related to Figure 2

At both timepoints (hospital admission and one month thereafter), Dirichlet multinomial mixture clustering of CAP patients resulted in two clusters: one with disrupted microbiota (n=55 at admission; n=44 after one month) and the other with undisrupted microbiota (n=60 at admission; n=40 after one month). Community composition (ß-diversity with weighted Unifrac distance) of CAP-patients with disrupted microbiota differed from patients with undisrupted microbiota and from controls, at hospital admission (A) and at one month (B).

Differentially abundant genera (Benjamini-Hochberg adjusted p-value <0.05 and ≥ 2 log2 fold-change) between clusters at admission (C) and one month following hospitalization (D). Chord diagram (E) showing the number of patients (represented by the size of the arrow) that remained within the same cluster between the two timepoints, or shifted between microbiota clusters (e.g. from the undisrupted cluster at admission to the disrupted cluster one month thereafter). Microbiota clusters are subdivided into groups based on the antibiotic exposure between hospital admission and one month thereafter.

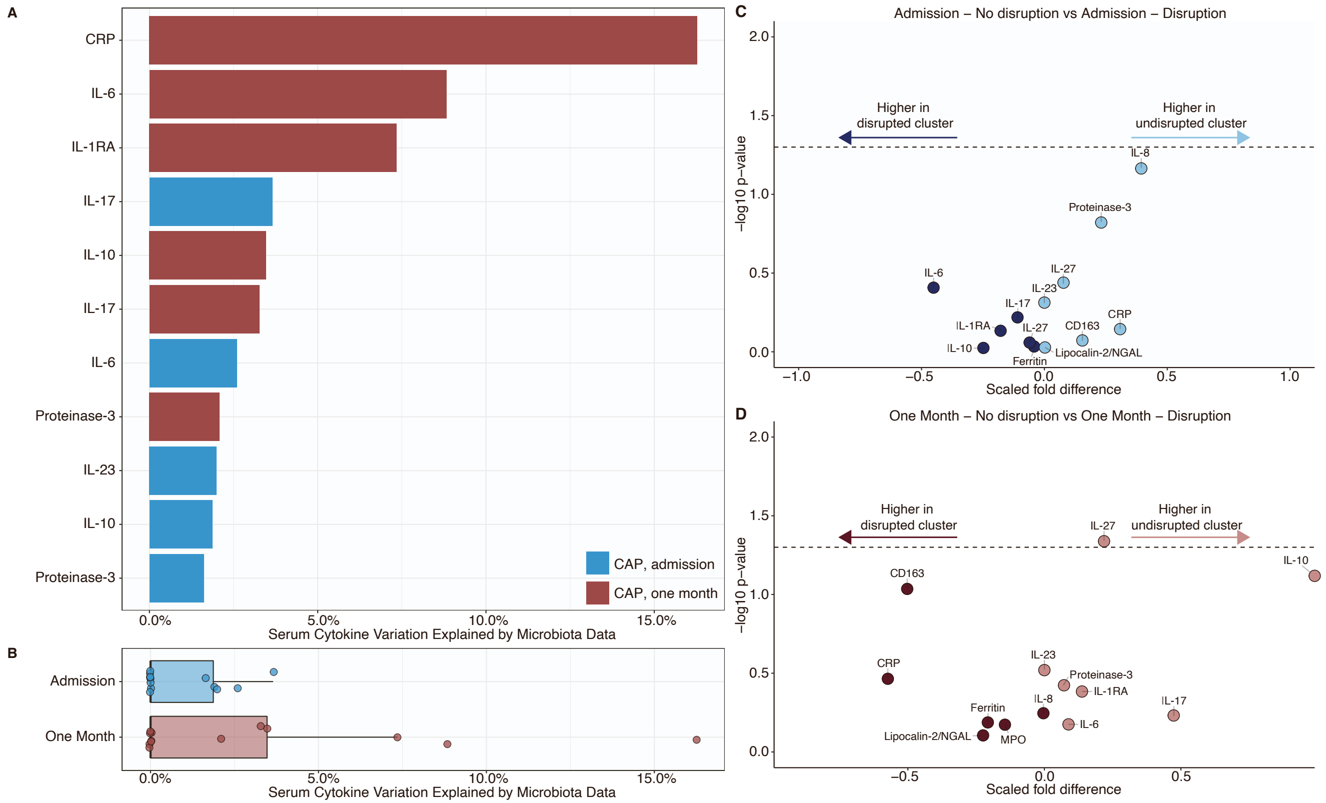

Figure S4. Relating gut microbiota to serum cytokine variation during and following CAP hospitalization, Related to Figure 3

(A) The largest percentage of serum cytokine variation explained by rectal microbiota was for cytokines at one month following hospitalization (CRP, IL-6, IL-1RA). (B) The amount of variation in serum cytokine responses and degranulation products explained by rectal microbiota did not differ between patients at hospital admission for CAP compared to patients at one month following hospitalization. Microbiota are represented by the first 4 principal coordinates (PCoA with weighted Unifrac distance) that explain ~65% of the variance, to avoid overestimation due to correlations between principal coordinates. The cytokine variance explained by these principal coordinates was estimated through permutation ANOVA by summing over the significant contributions ($p<0.2$). In the boxplot, dots represent the percentage of cytokine variation explained by microbiota data for each cytokine response (also shown in panel A), the central rectangle spans the first quartile to the third quartile (the interquartile range or IQR), the central line inside the rectangle shows the median, and whiskers above and below the box. (C) No significant differences ($p>0.05$) in cytokine responses between clusters A (n=55; undisrupted) and B (n=60; disrupted) at hospital admission. (D) One month following hospitalization, serum IL-27 was higher in patients with undisrupted microbiota profiles (n=40) compared to patients with disrupted microbiota profiles (n=44).

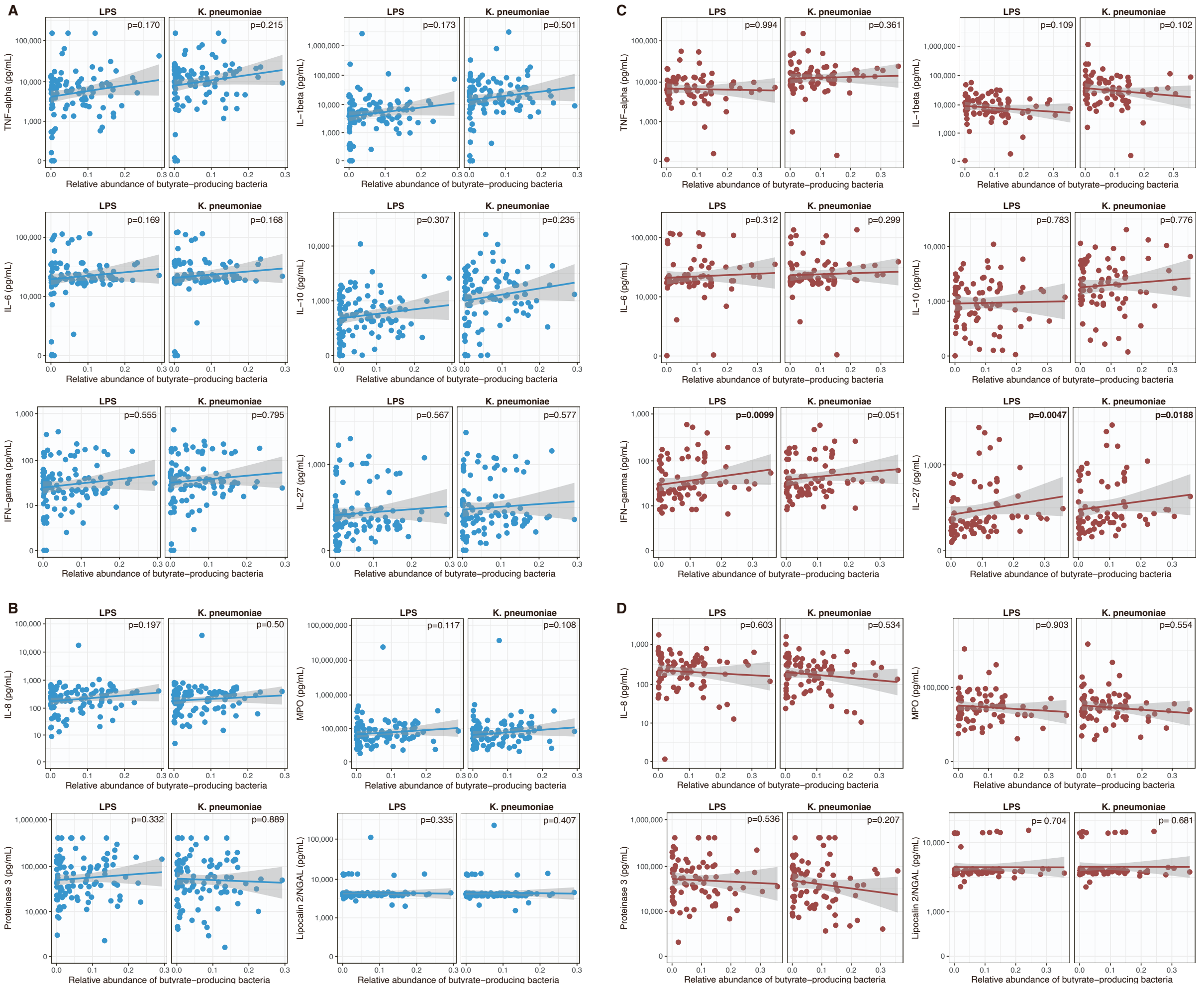

Figure S5. Correlation between abundance of butyrate-producing bacteria and cytokine production capacity of monocytes, Related to Figure 3

(A) At hospital admission, there was no relation between the relative abundance of butyrate-producing bacteria and the cytokine producing capacity of CD14+ monocytes upon *ex vivo* stimulation with LPS or heat-killed *K. pneumoniae*. (B) The cytokine producing capacity of polymorphonuclear cells (upon *ex vivo* stimulation with LPS or heat-killed *K. pneumoniae*) was not correlated with the ssrelative abundance of butyrate-producing bacteria.

(C) At one month following hospitalization, higher relative abundances of butyrate-producing bacteria were associated with higher levels of IFN-ɣ (upon LPS stimulation) and IL-27. (D) No relation between the relative abundance of butyrate-producing bacteria and cytokine measurements from polymorphonuclear cells at one month following hospital admission. Shaded areas indicate the 95% confidence interval. Statistical significance was determined by Spearman's correlation coefficient.

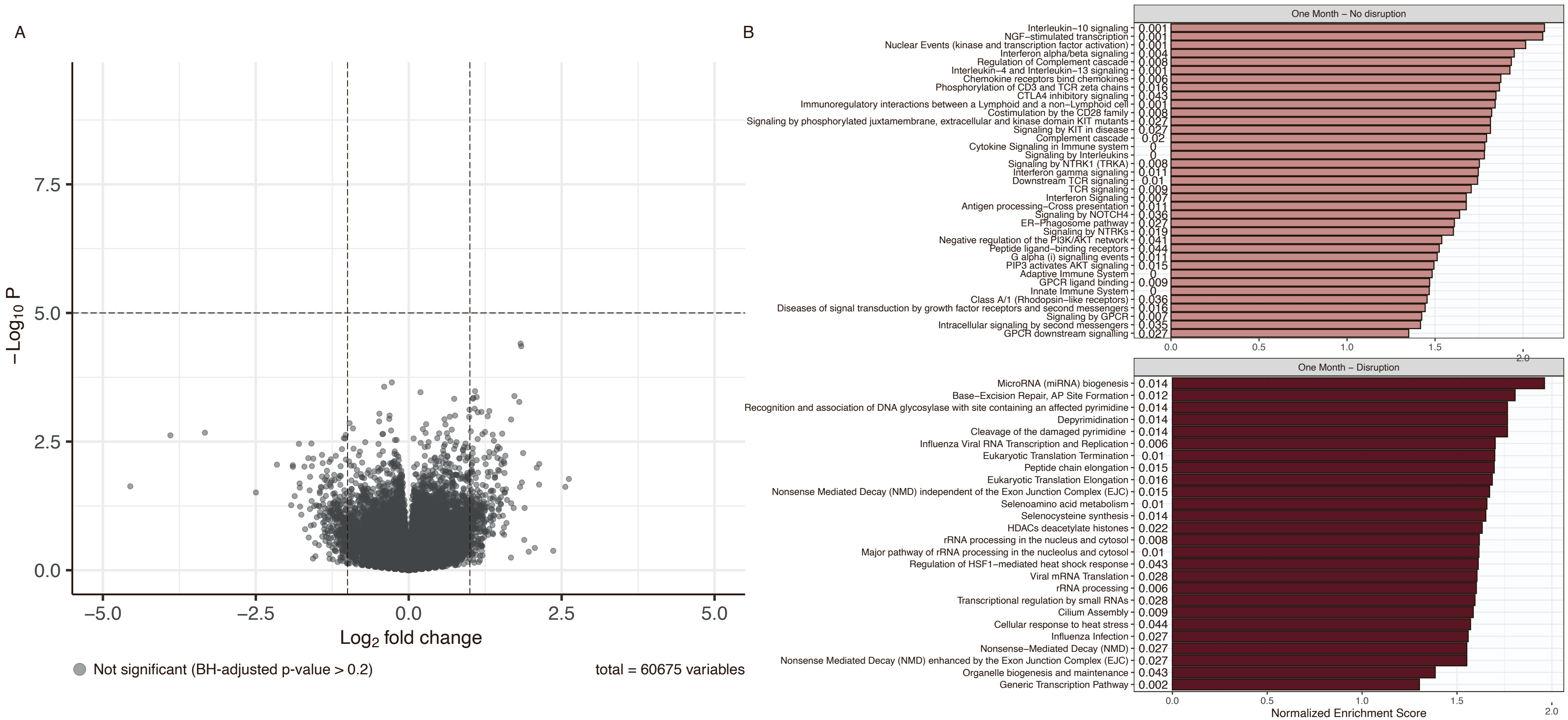

Supplemental Figure 6. Genome-wide transcriptional RNA profiling of patients one month following hospitalization

(A) Depiction of the Log2 fold changes and p-values of all individual gene transcripts (n=60,675) of monocytes isolated from CAP patients with undisrupted microbiota profiles relative to CAP patients with disrupted microbiota profiles, one month following admission. No gene transcripts breached Benjamini-Hochberg adjusted significance (**α**<0.2). (B) All significantly (Benjamini-Hochberg adjusted p-value <0.05) enriched pathways from the Functional Gene Set Enrichment Analysis of monocytes of patients with undisrupted microbiota profiles (n=27; top) and pathways enriched in monocytes of patients with disrupted microbiota profiles (n=26; bottom).
